# Inhibition of Histone H3 Lysine-27 Demethylase Activity Relieves Rheumatoid Arthritis Symptoms *via* Repression of IL6 Transcription in Macrophages

**DOI:** 10.3389/fimmu.2022.818070

**Published:** 2022-03-15

**Authors:** Zhan Zhao, Yazhuo Zhang, Danling Gao, Yidan Zhang, Wenwei Han, Ximing Xu, Qiaoling Song, Chenyang Zhao, Jinbo Yang

**Affiliations:** ^1^ School of Medicine and Pharmacy, Ocean University of China, Qingdao, China; ^2^ Innovation Platform of Marine Drug Screening & Evaluation, Qingdao Pilot National Laboratory for Marine Science and Technology, Qingdao, China

**Keywords:** H3K27me3, IL6, macrophage polarization, rheumatoid arthritis, epigenetic regulation, GSK-J4

## Abstract

Rheumatoid arthritis (RA) occurs in about 5 per 1,000 people and can lead to severe joint damage and disability. However, the knowledge of pathogenesis and treatment for RA remains limited. Here, we found that histone demethylase inhibitor GSK-J4 relieved collagen induced arthritis (CIA) symptom in experimental mice model, and the underlying mechanism is related to epigenetic transcriptional regulation in macrophages. The role of epigenetic regulation has been introduced in the process of macrophage polarization and the pathogenesis of inflammatory diseases. As a repressive epigenetic marker, tri-methylation of lysine 27 on histone H3 (H3K27me3) was shown to be important for transcriptional gene expression regulation. Here, we comprehensively analyzed H3K27me3 binding promoter and corresponding genes function by RNA sequencing in two differentially polarized macrophage populations. The results revealed that H3K27me3 binds on the promoter regions of multiple critical cytokine genes and suppressed their transcription, such as IL6, specifically in M-CSF derived macrophages but not GM-CSF derived counterparts. Our results may provide a new approach for the treatment of inflammatory and autoimmune disorders.

## Introduction

Rheumatoid arthritis (RA) is an aggressive immune mediated joint disease with synovial inflammation and joint destruction of extracellular matrix, which involves various immune cell populations, such as T cells, B cells and macrophages (1). In the pathogenesis of RA, macrophages synthesize cytokines and chemokines which are involved in the process of joint tissue damage (2). As the highly plastic cell populations, macrophages respond rapidly to many stimuli, making them potentially very important in the pathogenesis of RA (3). The myeloid colony stimulating factors GM-CSF and M-CSF are known to modulate the magnitude, duration, and character of many forms of inflammatory response (GM-CSF promotes M1 while M-CSF promotes M2) in mice and human macrophage populations by promoting distinct patterns of inflammatory cytokine/chemokine expression (4, 5). Moreover, GM-CSF had proven to be excessive secreted and contributed to autoimmune arthritis in RA patients (6). Therefore, it is important to study macrophage behaviors under the treatment of these growth factors.

Abnormal levels of cytokines were observed in serum and synovial tissue of early RA patients (7, 8). Both transcription factors and epigenetic mechanisms were shown to control macrophage activation and cytokine expression in RA (9). Epigenetic histone modification, such as methylation and acetylation, which is an intense research area in gene transcriptional regulation (10, 11), was shown to play a vital role in the process of macrophage differentiation (12). As the most common modified site, lysine residues in histones play a key role in gene activation or silencing (13, 14). Tri-methylation of lysine 27 on histone H3 (H3K27me3), a widely studied histone modification, represses gene transcription and subsequently influences cellular biological functions and tissue homeostasis. Researchers found that loss of H3K27me2/3 results in increased production of IL6 and IFNβ in thioglycolate elicited mouse peritoneal macrophages (15). Recent study revealed that *Leishmania donovani* down-regulated iNOS gene expression by influencing H3K27me3 repressive role in LPS and IFNγ stimulated J774 macrophages (16, 17), indicating a close relationship between H3K27me3 and gene expression in macrophage populations.

Methylation on H3K27 is reversible and can be dynamically regulated by site-specific histone methyltransferases and demethylases. Enhancer of zeste homolog 2 (EZH2), a methyltransferase, which is the component of polycomb repressive complex 2 (PRC2) complex, is the “writer” that leads to high level of H3K27me3. In contrast, demethylases such as Ubiquitously Transcribed tetratricopeptide repeat on chromosome X (UTX) and Jumonji domain containing protein 3 (JMJD3), is the “eraser” that specifically decreases di- and trimethylation on H3K27, thus lead to transcriptional activation (18). Recently, numerous studies suggested that H3K27me3 could function as an effector of some chronic diseases, such as tumorigenesis (19–21), osteoarthritis (22), liver fibrosis (23) and virus infection (24). It was also demonstrated that JMJD3 fine-tuned host response to streptococcus pneumoniae (25), and suppressing JMJD3 expression is likely to modulate the inflammatory response and reduce the progression of RA (26). Neele et al. reported that tissue specific knockout kdm6b (encoding JMJD3) in myeloid population resulted in advanced atherosclerosis (27), and macrophage kdm6b was shown to control the profibrotic transcriptome signature of foam cells (28). GSK-J4, a JMJD3/UTX inhibitor (29), has been reported to perform anti-inflammatory effect, such as anti-acute myeloid leukemia (30, 31), anti-schistosomal (32), and repressing the inflammatory colitis (33). It was reported that epigenetic alterations such as DNA methylation and histone modification might contribute to RA pathogenesis (26, 34), however the underlining molecular mechanisms remain largely unknown.

In the current study, our results revealed that demethylase inhibitor GSK-J4 could relieve the RA symptoms in Collagen Induced Arthritis (CIA) mice by reducing IL6 expression in macrophage populations. We further compared the levels of common epigenetic modifications in M-CSF and GM-CSF derived macrophage populations (M-BMDMs and GM-BMDMs), and identified H3K27me3 as the transcriptional suppressor in M-BMDMs specifically. GSK-J4 was found to repress gene transcription and NFκB binding activities in GM-BMDMs, in which the steady state level of H3K27me3 is quite low. Overall, these findings identify H3K27me3 as a critical regulator of monocyte/macrophage-mediated inflammation during RA progression and demonstrate GSK-J4 is feasible to reduce RA severity, which might shed light on the potential therapeutic application of macrophage epigenetic regulators in RA patients.

## Materials and Methods

### Animals

Eight weeks C57BL/6J and DBA/1J male mice were purchased from Beijing Vital River Laboratory Animal Technology Co., Ltd. Mice were housed under a 12-hour light/dark cycle in a temperature-controlled room (22°C) with free access to water and food. C57BL/6J mice were used for BMDM isolation, and DBA/1J mice were used for CIA model. All mice were maintained and used with the approval of the Committee of Experimental Animals of the Ocean University of China (OUC-SMP-2018-12-02) and conformed to the Guide for the Care and Use of Laboratory Animals published by the United States National Institutes of Health (NIH Publication No 85-23, revised 1996).

### Cell Culture and Treatment

Human monocytic THP-1 cells were cultured in RPMI 1640, supplemented with 10% low-endotoxin fetal bovine serum (VivaCell, Shanghai, China), 1% Pen/Strep, and differentiated into macrophage by incubation with 100 nM Phorbol 12-myristate 13-acetate (PMA) for 72 hours.

Details of primary BMDM isolation have been described (35). After centrifugation and cell counting, BMDM were seeded in 60 mm cell culture dishes with 5 ml DMEM (supplemented with 10% low-endotoxin fetal bovine serum, 1% Pen/Strep) and differentiated with either 20 ng/ml GM-CSF or 50 ng/ml M-CSF (R&D) for 7 days.

For the experiments with inhibitors, DZNeP (Selleck, S7120) or GSK-J4 (TargetMol, T22819) were added into medium on the 5^th^ day of growth factors mediated differentiation at the final concentration of 10 μM or 30 μM, respectively and the cells were further cultured for 48 hours. LPS (100 ng/ml) was then used to stimulate BMDM for indicated time before the sample collections.

### RNA Isolation and Real-Time qPCR Analysis

Cells were lysed and RNA was extracted using TRIzol reagent. One microgram total RNA was reverse transcribed into cDNA using One Step PrimeScript™ RT-PCR Kit (Takara) according to the manufacturer’s instructions. The real-time PCR reaction was carried out on StepOne Plus (Vazyme, Cat. Q511-02) and the primer sequences were listed in **Table 1**. The PCR was carried out for 40 cycles of 95°C for 30 s, 58°C for 30 s, and 72°C for 45 s, and final elongation at 72°C for 5 minutes. The relative gene expression level was normalized to that of GAPDH in the same sample. Details of qPCR analysis have been described previously (36).

**Table 1:** qPCR primer sequences.

Gene	Forward Sequence	Reverse Sequence
IL6	TAGTCCTTCCTACCCCAATTTCC	TTGGTCCTTAGCCACTCCTTC
IL6 (TSS)	TGTAGACCATGTAGTTGAGGTCA	AGGGCAAGGAACTGCCTT
IL6 (952 bp)	CAGTGGGATCAGCACTAACAGA	CCATTAGAAACAACTGGTCCTGAC
IL10	GCTCTTACTGACTGGCATGAG	CGCAGCTCTAGGAGCATGTG
IL12b	TGGTTTGCCATCGTTTTGCTG	ACAGGTGAGGTTCACTGTTTCT
TNF	CCCTCACACTCAGATCATCTTCT	GCTACGACGTGGGCTACAG
IL1b	GCAACTGTTCCTGAACTCAACT	ATCTTTTGGGGTCCGTCAACT
GAPDH	AGGTCGGTGTGAACGGATTTG	TGTAGACCATGTAGTTGAGGTCA

### RNA-seq

The mRNA of BMDMs were extracted using TRIzol, then reversed transcribed to make the cDNA library. The cDNA library was then sequenced on an Illumina platform. Genes with corrected p value < 0.05 and |log2 (Fold Change)| >1 were assigned as significant differentially expressed genes (DEGs). Gene Ontology (GO) enrichment and Kyoto Encyclopedia of Genes and Genomes (KEGG) pathways analysis of DEGs were conducted by DESeq. Gene set enrichment analysis (GSEA) was performed on GSEA 4.1.0 according to the instructions provided on the GSEA website (https://www.gsea-msigdb.org/gsea/index.jsp).

### Small Interfering RNA

BMDM were transfected with 20 nM of small interfering (siRNA) against UTX or JMJD3 by Lipofectamine™ RNAiMAX Transfection Reagent (Invitrogen) according to the manufacturer’s instructions. Forty-eight hours post transfection, BMDM were used for LPS treatment.

### ChIP

Chromatin immunoprecipitation (ChIP) assay was carried out by using a ChIP assay kit (Cell Signaling Technology, Cat.9003), according to the manufacturer instructions and our previous experience (37). Briefly, cells were fixed with 1% formaldehyde in medium, then washed with phosphate-buffered saline (PBS), lysed and sonicated in ChIP buffer (Cell Signaling Technology, Cat.7008). Cell lysate was then immunoprecipitated with rabbit anti-H3K27me3 (Active Motif, Cat.39535), anti-H3K4me3 (Active Motif, Cat.61379) or anti-RNAPII (Active Motif, Cat.39497) antibodies. After de-fixation, the H3K37me3 bound DNA sequences were quantified by real-time PCR as described above, the primer sequences were listed in **Supplementary Table 1**.

### Immunoblotting

Cells were lysed in lysis buffer (Cell Signaling Technology Cat.9803), briefly sonicated, kept on ice for 30 minutes, and centrifuged at 15,000 g for 15 minutes. The protein concentration in supernatant was quantified by BCA assay kit (Beijing Solarbio Science & Technology Co., Ltd.). Loading buffer were then added into 20-30 μg total proteins, heated at 95°C for 5 minutes, then cell lysates were resolved in 8%-12% SDS-PAGE. The proteins were then transferred onto a nitrocellulose membrane (Invitrogen). After overnight incubation with appropriate primary antibody at 4°C, the stripes were incubated with HRP linked second antibody for 1 hour at room temperature, then visualized with ECL western blotting substrate. The primary antibodies used in this study include: anti-EZH2 (CST, Cat.5246), UTX (CST, Cat.33510), JMJD3 (Absin, Cat.abs105155), GAPDH (Aksomics, Cat.KC-5G4), H3K27Ace (CST, Cat.8173), H3K4me (Active Motif, Cat.39635), H3K9me (Active Motif, Cat.39681), H3K20me (Active Motif, Cat.39027), H3K27me3 (Active Motif, Cat. 39535), and H4K20me3 (Active Motif, Cat.39672).

### ELISA

The level of IL6 in the supernatants of cultured cells post LPS challenge were measured. For mice serum, peripheral blood was centrifuged at 1,000 g for 15 minutes before serum collection. For mice tissues, 10 mg tissues were isolated and grinded in 1.0 ml lysis buffer. After centrifugation at 1,000 g for 15 min, the supernatants were collected. The levels of IL6 (Animal union, Shanghai, China), IL10, TNFα (Cloud-clone) and IL17 (ExCell Bio) were measured by enzyme linked immunosorbent assay (ELISA) according to the manufacturer instructions.

### Flow Cytometry

Cells in lymph gland were isolated to make single cell suspension. Cells were blocked with blocking buffer (20% FBS, 1:100 CD16/CD32 antibodies and 1:100 Rat IgG) for 20 minutes and then stained with fluorescence conjugated antibodies for 30 minutes at 4°C. The samples were washed with FACS buffer (PBS, 0.5-1% BSA or 5-10% FBS, 0.1% NaN3 sodium azide), acquired and analyzed by FACS Arial III (BD Biosciences). The FlowJo software was utilized for data analysis (Tree Star, San Carlos, Calif).

### HE Staining and Immunofluorescence

Arthrosis tissues were fixed in 4% paraformaldehyde and embedded in paraffin and then stained with hematoxylin-eosin (H&E). For immunofluorescence, sections were deparaffinized, inactivated peroxidase, and after antigen retrieval, blocked in PBST containing 10% goat serum and incubated with primary antibodies against IL6 (CST, Cat.12912), H3K27me3 (Active Motif, Cat.39535) and CD11b (Santa cruze, Cat.sc-1186) overnight at 4°C. Sections were then counterstained with DAPI and examined using a Zeiss microscope. Randomly selected fields of ten views per mice, and the positive cell fluorescence intensity was calculated using Image J software. Six mice per group were analysed.

### Lactate Dehydrogenase Assay

The media of GSK-J4 treated or siRNA transfected BMDMs were collected and centrifuged at 500 g for 10 mins. The lactate dehydrogenase (LDH) concentration in supernatants was detected using a LDH assay kit (NJJCBIO, China). Detailed experimental procedures have been described elsewhere (36).

### EMSA and NFκB Binding Activity Measurement

Nuclear extracts from BMDMs were prepared by high salt extraction (36). Electrophoretic Mobility Shift Assays (EMSA) were conducted by using EMSA/Gel shift kit (Beyotime) with the standard method as described previously (38). The NFκB consensus oligo sequence is 5′-AGT TGA GGG GAC TTT CCC AGG C-3′ and 5′- CGG ACC CTT TCA GGG GAG TTG A -3′.

NFκB binding activity was measured using TransAM DNA binding ELISAs (Active Motif) following the manufacturer instructions. Quantitative data were analyzed with indicated statistical tests and visualized in R, Prism, or Excel software.

### Statistical Analyses

Data is represented graphically as the mean ± SD. Statistical analyses were performed using Prism version 8. Paired or Unpaired t-test, One- or Two- way ANOVA were used for calculating p values as indicated in each figure legend. p < 0.05 was considered statistically significant.

## Results

### Demethylase Inhibitor GSK-J4 Ameliorates Collagen Induced Arthritis *In Vivo*

**Figure 1:**
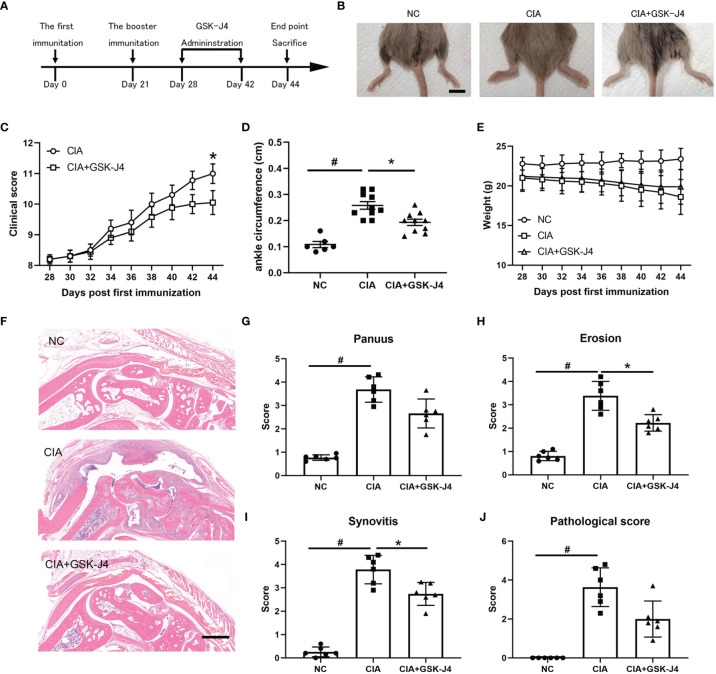
Demethylase inhibitor GSK-J4 ameliorates collagen induced arthritis (CIA) *in vivo.* **(A)** The flow diagram of CIA model and GSK-J4 treatments. **(B)** Therapeutical effect of GSK-J4 on DBA1 mice. **(C)** According to joint swelling extend, scored 0~5 to all the paws. The scale bars represent 100 μm. **(D)** Ankle circumferences were measured at the 44th day. **(E)** Mice body weights were measured every two days after GSK-J4 administration. **(F)** HE staining of the hind legs of CIA mice, scale bar represents 100 μm. **(G–I)** Pathologic score of pannus, erosion and synovitis. **(J)** Pathological score of each given groups. p < 0.05 (two-tailed Student’s T-test) is indicated by * for comparison with CIA vs. CIA+GSK-J4, # for comparison with NC vs. CIA, error bars indicate SD.

Abnormal secretion of IL6 and other cytokines have been found to play a critical role in RA pathology (39, 40). Using well established CIA models, we studied whether demethylase inhibitor GSK-J4 administration could relieve RA symptoms by reducing cytokine levels, such as IL6. CIA models and GSK-J4 administration were executed following the flowchart shown in **Figure 1A**. Sixteen days GSK-J4 treatment relieved CIA induced joint swelling (**Figure 1B**), reduced clinical score (**Figure 1C**) and the ankle circumference (**Figure 1D**), with limited influence on mice body weight (**Figure 1E**). Mice hind legs were decalcificated and fixed by paraformaldehyde, then visualized by HE staining (**Figure 1F**). There are significant amounts of immune infiltration in the synovial cavities of CIA mice, which were largely reduced in GSK-J4 treatment group. In addition, the synthesis of pannus, erosion, synovitis and pathological score (41) was evaluated. The results clearly showed that the reduced severity of joint upon GSK-J4 treatments (**Figures 1G–J**). Taken together, our results suggested that GSK-J4 could exert therapeutical effects on CIA mice.

### GSK-J4 Reduces H3K27me3 Level and Cytokine Expression in Macrophages in CIA Mice

**Figure 2:**
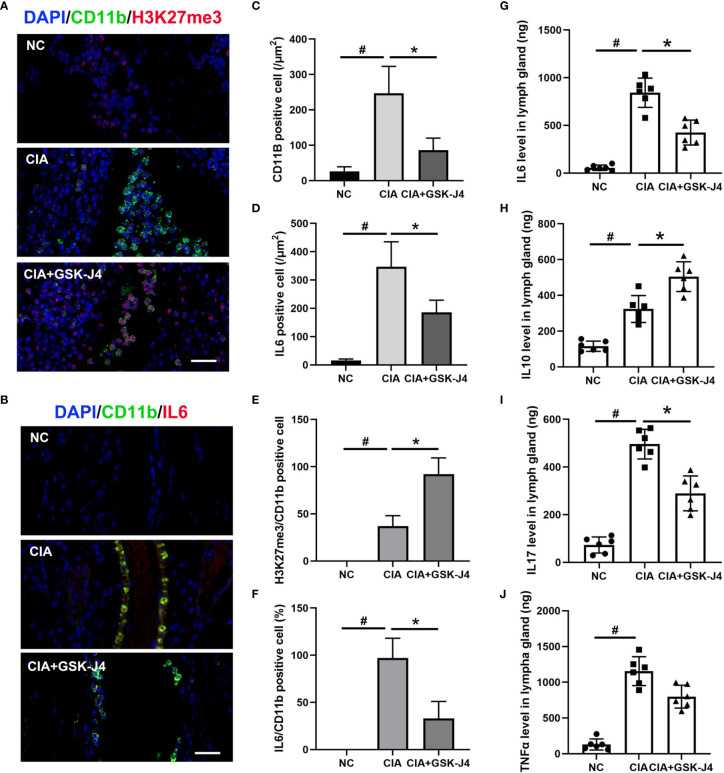
GSK-J4 reduces H3K27me3 level and cytokine expression in macrophages in CIA mice. **(A)** Immunofluorescence staining of H3K27me3 (red) and CD11b (green) positive cells on CIA mice joints, the scale bars represent 100 μm. **(B)** Immunofluorescence staining of IL6 (red) and CD11b (green) positive cells, the scale bars represent 100 μm. **(C, D)** Average fluorescence intensity of IL6 and CD11b positive cells. **(E, F)** The proportion of H3K27me3 and IL6 in CD11b positive cells. **(G–J)** The concentration of IL6, IL10, IL17 and TNFα in mice lymph gland were detected by ELISA. p < 0.05 (two-tailed Student’s T-test) is indicated by * for comparison with CIA vs. CIA+GSK-J4, # for comparison with NC vs. CIA, error bars indicate SD.

To verify whether H3K27me3 in macrophages plays a vital role in GSK-J4 treated CIA mice, the slides of mice hind legs were stained for IL6, H3K27me3 and CD11b by immunofluorescence. There were at least two distinct CD11b positive macrophages, CD11b+H3K27me3+ and CD11b+IL6+, were found between cartilago articularis and synovial membrane of CIA mice. Upon CIA modeling, the level of H3K27me3 significantly reduced, which could be reversed by GSK-J4 administration (**Figure 2A**). Consistently, the amounts of CD11b+, IL6+ and CD11b+IL6+ cells increased in CIA mice, but decreased after 16 days GSK-J4 treatment (**Figures 2B–F**), suggesting that GSK-J4 could reduce the levels of H3K27me3+ and IL6 in macrophage populations. Furthermore, decreased levels of IL6, IL10, IL17 and TNFα in mice inguinal lymph glands were observed after treatment with GSK-J4 (**Figures 2G–J**), which was consistent with the reduced local inflammation. Moreover, systemically decreased levels of IL6 and TNFα were also observed in peripheral blood (**Figure S1**). As altered IL17 and IL10 levels were observed, which are crucial in Th17 and Treg differentiation and Th17 and Treg were reported to play indispensable roles in RA (42–44), the abundance of CD4+IL17+ and CD4+ CD25+FOXP3+ cells in mice inguinal lymph node was evaluated by flow cytometry. The ratio of Treg/Th17 has been used to characterize the severity of RA (45, 46), and our results showed that GSK-J4 could downregulate the ratio of Treg/Th17 (**Figure S2**), further suggesting that inhibition of demethylase activity could be beneficial to CIA, at least in the experimental RA mice model.

### GSK-J4 Inhibits Cytokine Expression in Macrophage

**Figure 3:**
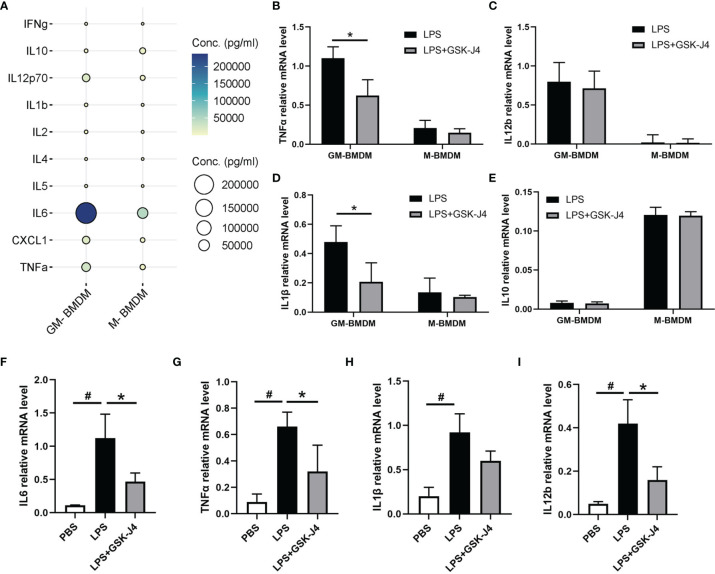
GSK-J4 inhibits cytokine expression in macrophages. **(A)** The expression of cytokines in the culture media of BMDMs. **(B–E)** BMDMs were treated with GSK-J4 for 48 hours, the RNA levels of TNFα, IL12b, IL1β and IL10 were determined by qRT-PCR. **(F–I)** THP-1 cells were firstly differentiated with PMA for 72 hours and followed by treatment with GSK-J4 for 48 hours, the RNA levels of IL6, TNFα, IL1β and IL12b were measured by qRT-PCR. p < 0.05 (two-tailed Student’s T-test) is indicated by * for comparison with LPS+GSK-J4 vs. LPS, # for comparison with NC vs. LPS, error bars indicate SD.

Numerous studies have demonstrated that GM-CSF (GM-BMDM) and M-CSF (M-BMDM) derived macrophages exert quite different phenotypes, which are partially represented by their different gene expression patterns (4, 47–49). Moreover, the high level of GM-CSF was shown to be associated with RA (5, 50–52), while M-CSF is the default macrophage growth factor almost in all tissues (53–55). Therefore, we investigated the response of GM-BMDM and M-BMDM to GSK-J4 in order to better understand the mechanisms underlying the protective effects of GSK-J4 for CIA mice. First of all, we measured cytokine expression patterns in GM-BMDMs and M-BMDMs post LPS stimulation. The results showed distinct cytokine expression profiles in these macrophage populations, and some pro-inflammatory cytokines, such as TNFα, IL12p70 and IL6 exhibited the significantly high levels in GM-BMDMs, with IL6 being the most dominant one (**Figure 3A**). Not only IL6, other cytokines or chemokines, such as IL1β and IL12b, showed higher expression in GM-BMDMs as well, and the RNA expression of TNFα and IL1β was reversed by GSK-J4 (**Figures 3B–E**). Quite interestingly, the expression levels of IL6, TNFα and IL1β were also influenced by GSK-J4 in human monocyte THP-1 cells challenged with LPS (**Figures 3F–I**), which indicated that GSK-J4 regulated cytokine gene expression may also operate in human macrophages. To sum up, GSK-J4 could alter cytokine gene expression in GM-BMDMs.

### GSK-J4 Inhibits IL6 Expression *via* Regulating H3K27me3 Activity in Macrophage

**Figure 4:**
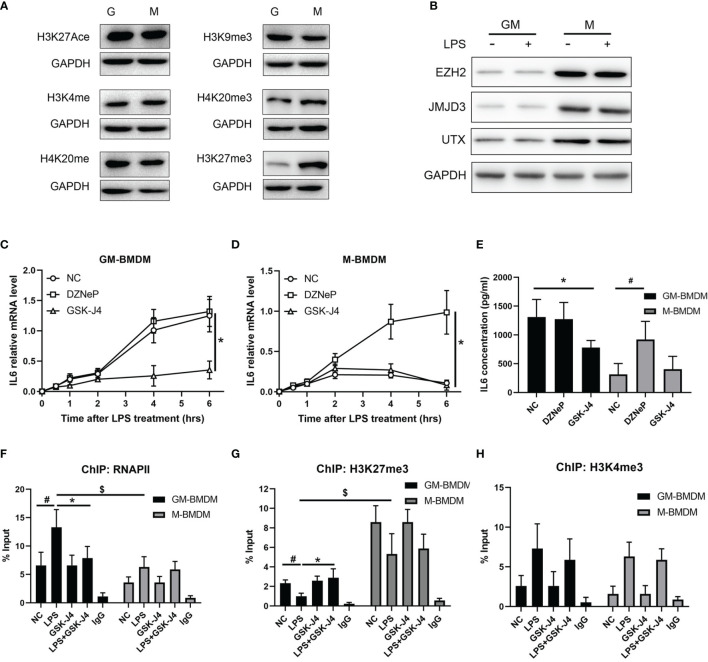
GSK-J4 inhibits IL6 expression *via* regulating H3K27me3 activity in macrophages. **(A)** Levels of epigenetic modifications (H3K27Ace, H3K4me3, H4K20me, H3K9me3, H4K20me3, H3K27me3) were detected in GM-BMDMs and M-BMDMs. **(B)** BMDMs were stimulated with 100 ng/ml LPS for 2 hours, the expression levels of EZH2, JMJD3 and UTX were determined by Western Bot. **(C, D)** After exposing to DZNeP and GSK-J4 for 48 hours, qPCR determines IL6 mRNA level changes upon LPS stimulation in GM-BMDMs **(C)** and M-BMDMs **(D)**. **(E)** GM-BMDMs and M-BMDMs were stimulated with LPS (100 ng/ml) for 6 hours, the concentration of IL6 in the media were measured by ELISA. ChIP experiments were performed with anti-RNAPII **(F)**, anti-H3K27me3 **(G)** and anti-H3K4me3 **(H)**, the abundance of IL6 promoter fragment (-1000 to -900 bps relative to TSS) was determined by qPCR. p < 0.05 (two-tailed Student’s T-test) is indicated by * for comparison with LPS+GSK-J4 vs. LPS, # for comparison with NC vs. LPS, $ for comparison DZNeP vs. NC under LPS stimulation, error bars indicate SD.

To further analyze the mechanism of GSK-J4 regulation of IL6 expression in macrophages, a variety of common epigenetic modifications that have either enhanced or inhibited roles on transcription, including H3K27Ace, H3K4me3, H4K20me, H3K9me3, H4K20me3, H3K27me3 were evaluated in M-BMDMs and GM-BMDMs. Interestingly, the level of H3K27me3 was significantly different in these two populations (**Figure 4A**) with dominant expression in M-BMDMs. Interestingly, we found one locus -1000 to -900 bps upstream of TSS on IL6 promoter only exists in M-BMDMs (**Figure S3**). Similar to H3K27me3, its “writer” methyltransferase EZH2 and “eraser” demethylases (UTX and JMJD3) were found to be dominantly expressed in M-BMDMs, and 2 hours LPS stimulation cannot influence their expression (**Figure 4B**), suggesting that these differences mainly originated from 7 days GM-CSF and M-CSF treatments. Consistent with this observation, we previously found that IL6 expression was largely repressed in M-BMDMs comparing with GM-BMDMs (52). To further verify if H3K27me3 modification is critical to regulating IL6 expression, both GM-BMDMs and M-BMDMs were cultured at the presence of EZH2 inhibitor (10 μM DZNeP) or UTX/JMJD3 inhibitor (30 μM GSK-J4) to modulate the level of H3K27me3. Of note, GSK-J4 inhibited IL6 mRNA level in GM-BMDM (**Figure 4C**), and DZNeP increased IL6 mRNA level in M-BMDM (**Figure 4D**). IL6 protein levels determined by ELISA with the media from LPS stimulated GM- or M-BMDMs showed the similar results (**Figure 4E**). Next, ChIP assay were applied to analyze the binding of RNAPII and H3K27me3 on IL6 promoter. As expected, GSK-J4 reversed RNAPII binding activity of IL6 promoter in GM-BMDMs (**Figure 4F**), and H3K27me3 bound on IL6 promoter much less in GM-BMDMs than in M-BMDMs. Besides, increased H3K27me3 level by GSK-J4 could reverse LPS induced binding loss of H3K27me3 to IL6 promoter in GM-BMDM (**Figure 4G**). To rule out the possibility that GSK-J4 might stimulate transcription independent of H3K27me3, the parallel H3K4me3 ChIP assay were conducted in **Figure 4H**. We found that GSK-J4 influenced little on H3K4me3 binding activity, whose methylation usually correlate with transcriptional enhancement. Collectively, these data suggest that GM-CSF and M-CSF polarized macrophages exhibits different epigenetic modification profiles, which might contribute to their different gene expression patterns (4). Among the tested, H3K27me3 showed most distinct expression levels in two macrophage populations, which were also the case for its “writer” and “eraser”. Besides, H3K27me3 could alter IL6 expression by binding on its promoter regions and further inhibiting transcription.

### H3K27me3 Influences Gene Transcription *via* Reducing NFκB Binding Activity

**Figure 5:**
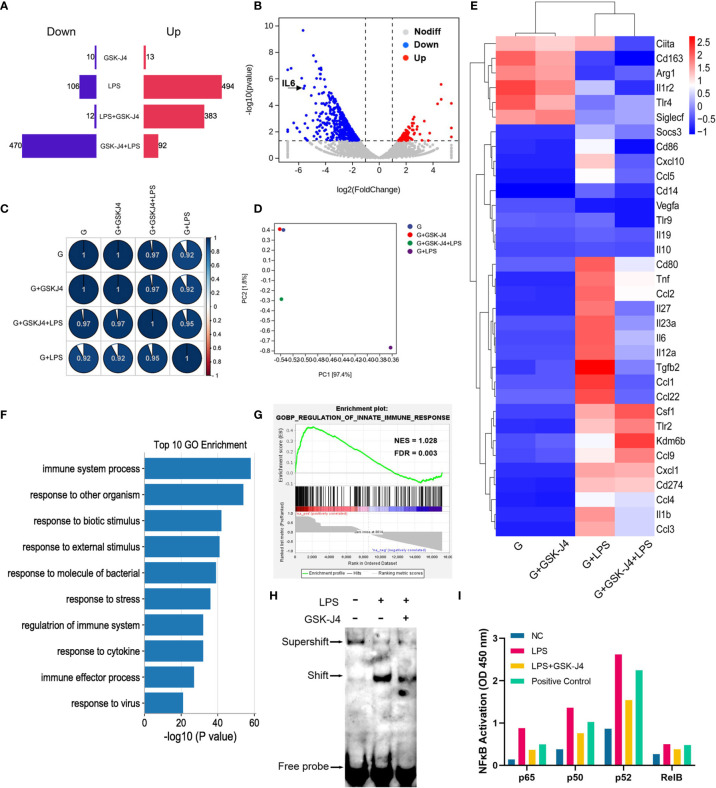
H3K27me3 influences gene transcription *via* reducing NFκB binding activity. **(A–G)** GM-BMDMs were treated with GSK-J4 for 48 hours (GSK-J4), or LPS for 1 hours (LPS), or pretreat with GSK-J4 for 48 hours then LPS for 1 hour (GSK-J4+LPS), or GSK-J4 and LPS co-stimulation for 1 hour (LPS+GSK-J4). After treatments, the cells were harvested, and the RNA were used for RNA-seq. **(A)** Amount of up (red) and down (blue) regulated genes, GSK-J4 stimulated for 48 hours or co-stimulated with LPS for 1 hour. **(B)** Volcano diagrams represent the distribution of DEGs in LPS vs. GSK-J4+LPS groups in GM-BMDMs. **(C)** Gene expression correlation index after normalization. **(D)** PCA analyses of indicated treatment groups in GM-BMDMs. **(E)** Heatmap of portion of immune related genes in the indicated treatment groups in GM-BMDMs. **(F)** Top 10 biology processes of GO terms with DEGs of LPS vs. GSK-J4+LPS groups. **(G)** GSEA analyzed Top one GSEA plot of LPS vs. GSK-J4+LPS. **(H)** EMSA assay detected NFκB probe binds to nucleus samples of LPS stimulated GM-BMDM with or without GSK-J4 treatment. **(I)** GM-BMDMs were left untreated (NC), LPS treated for 1 hour, or LPS+GSK-J4 co-treated for 1 hour. The cells were harvested, and the nuclei were isolated. The nuclear lysis was then applied to the ELISA plate coated with the antibodies against different NFκB subunits. Binding activities of these samples on p65, p50, p52, Rel B subunits were then detected, Raji samples provided by kit worked as the positive control.

It is not difficult to speculate that H3K27me3 should inhibit a set of genes’ transcription besides IL6. To verify this hypothesis, GM-BMDMs were stimulated with LPS, and GSK-J4 was used to evaluate the contribution of H3K27me3 on gene expression *via* RNA sequencing analysis. After comparison with the reference genome, all the genes were filtrated and DEGs were identified. GSK-J4 was shown to be able to significantly down-regulate LPS stimulated genes. However, GSK-J4 itself has limited effects on transcriptome (**Figures 5A–D**). Further, the relative expression levels of some immune related genes, such as IL6, TNF, IL23a, etc. were influenced by GSK-J4 only upon LPS stimulation (**Figure 5E**). To explore the function of these DEGs, We compared the differences in biological functions before and after GSK-J4 administration by GO analysis and screened the top 10 biology processes (**Figure 5F**). As a result, most of them links to immune responses to stimuli. Enrichment plot “Regulation of Innate Immune Response” ranked the top1 in Gene Set Enrichment Analysis (GSEA) (**Figure 5G**), and other immune regulation gene sets were also ranked top 4 in the gene matrix downloaded from *ftp.broadinstitute.org://pub/gsea/gene_sets/c5.bp.v6.0.symbols.gmt* (**Figure S4**). NFκB is known to be the major transcription factor for IL6 (56, 57), therefore we speculate that modulation of H3K27me3 by GSK-J4 might influence NFκB activities. By applying EMSA assay with NFκB consensus probe, we found that GSK-J4 treatment could significantly alter NFκB binding activities (**Figure 5H**). Further assay on the activity of four different NFκB subunits showed that p65 and noncanonical pathways subunits p50 and p52 were actively translocated into the nucleus under GSK-J4 treatments. In contrast, activity of Rel B changed little when stimulated by LPS or GSK-J4 (**Figure 5I**). In summary, demethylase inhibitor GSK-J4 could reduce LPS stimulated gene transcription by reducing NFκB binding activities in GM-BMDMs.

### JMJD3 Regulates Macrophage Survival and Is the Major Contributor to H3K27me3 Mediated IL6 Transcription

**Figure 6:**
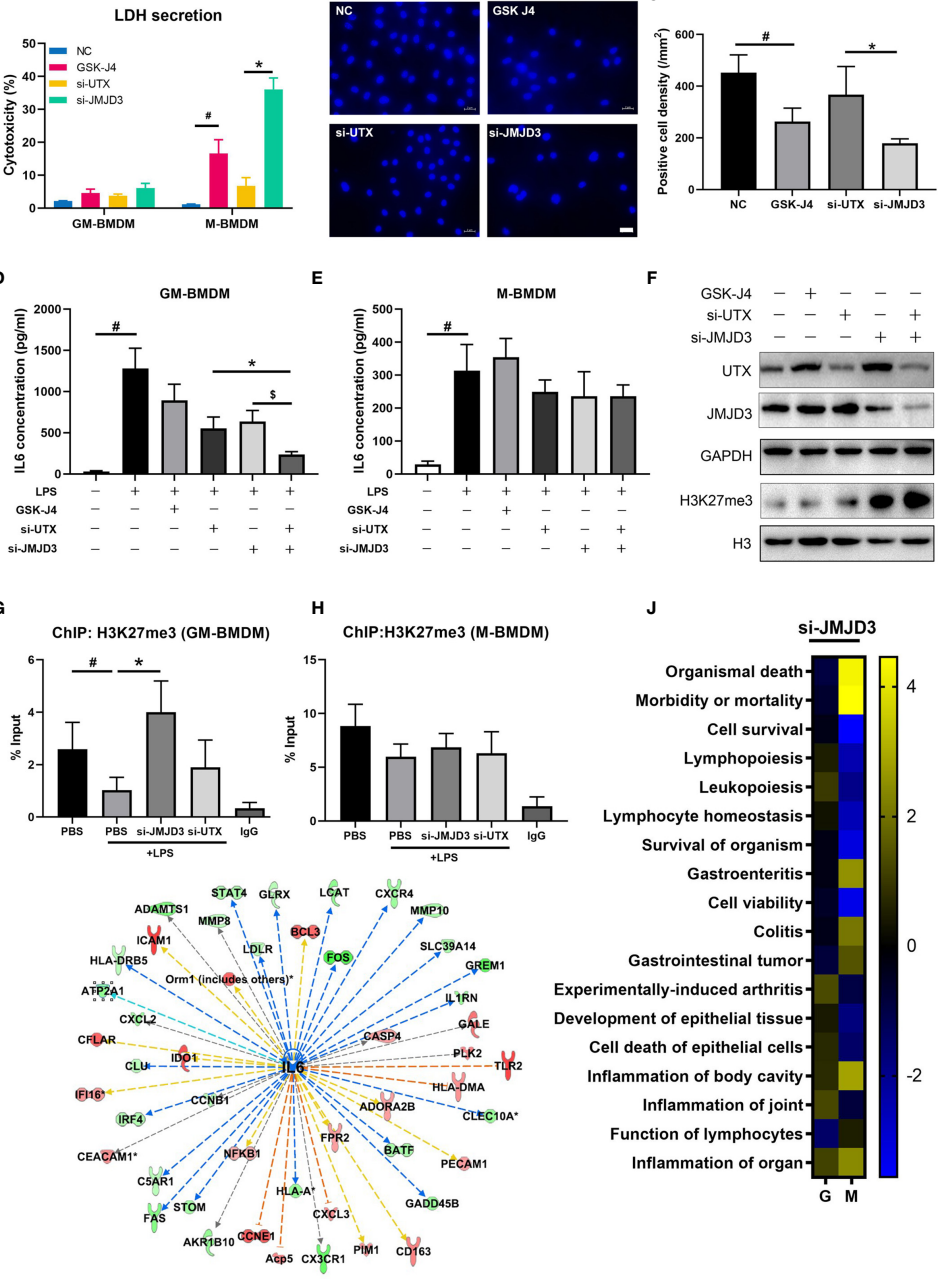
JMJD3 regulates macrophage survival and is the major contributor to H3K27me3 mediated IL6 transcription. **(A)** GM-BMDMs and M-BMDMs were left untreated (NC), or treated with GSK-J4 for 48 hours, or transfected with UTX siRNAs (si-UTX) or JMJD3 siRNAs (si-JMJD3) for 48 hours. The culture media were collected and the LDH concentration in the media were determined. **(B)** Representative images from M-BMDMs prepared as in **(A)** and stained with DAPI, white scale bar represents 50 μm. **(C)** Quantification of slides prepared as in **(B)** (5 slides/treatment group). **(D, E)** GM-BMDMs and M-BMDMs were treated as in **(A)**, and the IL6 concentrations in the media were detected by ELISA. **(F)** GM-BMDMs were treated as in **(A)**, and cell lysates were used to detect the protein levels of UTX, JMJD3 and H3K27me3. **(G, H)** GM-BMDMs or M-BMDMs were treated with PBS for 48 hours (PBS), or PBS 48 hours followed by LPS 1 hour (PBS+LPS), or transfected with UTX siRNAs (si-UTX) or JMJD3 siRNAs (si-JMJD3) for 48 hours. The cell nuclei lysates were used for H3K27me3 ChIP assays. After de-crosslinking, the H3K27me3 binding DNA fragment were examined for IL6 promoter (-952 to -1008) enrichment. **(I)** IPA analyzed RNA-seq data of si-JMJD3 transfected GM-BMDMs, and summarized the top upstream regulator, the detailed information of IL6 was shown. **(J)** IPA analyzed the activation scores of different signaling pathways in LPS (100 ng/ml for 1 hour) stimulated, si-JMJD3 transfected GM-BMDMs or M-BMDMs. Error bars indicate SD, p < 0.05 (two-tailed Student’s T-test) is indicated by * for comparison with si-UTX vs. si-JMJD3, # for comparison with NC vs. LPS, $ for comparison with si-JMJD3 vs. si-JMJD3 and UTX.

Previous reports have described that inhibition of JMJD3 can reduce proliferation and promote apoptosis of tumor cells (53), and STAT3-JMJD3 signaling is involved in macrophage death (54). In our experiment, we observed an increased mortality in M-BMDMs upon GSK-J4 treatment or knock down of H3K27me3 demethylases by transfection of UTX/JMJD3 siRNAs (**Figure 6A**). Upon GSK-J4 treatment, 18.6% cell death was observed in M-BMDMs, while si-JMJD3 treatment could increase the death rate to 32.2%. However, neither GSK-J4 nor si-JMJD3 exerted obvious cytotoxicity to GM-BMDMs. Surprisingly, it was not the case when UTX was silenced. Therefore, we considered that the toxic effect of si-UTX was trivial comparing to si-JMJD3 in M-BMDMs. Similar results were observed by DAPI staining (**Figures 6B, C**). These results suggested that JMJD3 played a more essential role in macrophage survival than UTX. And this observed cell death seems not because of induced apoptosis, as the expression levels of pro-Caspase3 and Bcl2 had no obvious changes after GSK-J4 treatment (**Figure S5**). Moreover, knock down of either UTX or JMJD3 could reduce IL6 secretion, simultaneously silencing of both could further lower IL6 expression only in GM-BMDMs but not in M-BMDMs (**Figures 6D, E**), indicating both UTX and JMJD3 have non-redundant roles in regulating IL6 expression. Interestingly, silencing UTX could reduce JMJD3 expression and vice versa (**Figure 6F**). By analyzing the H3K27me3 binding DNA fragments, we found that only in GM-BMDMs, knock down of JMJD3 but not UTX, reversed H3K27me3 binding activity on IL6 promoter (**Figures 6G, H**), indicating that the therapeutical effect of GSK-J4 mainly depends on its inhibition to JMJD3. Ingenuity Pathway Analysis (IPA) was used to predict the potential upstream regulators by analyzing the gene expression profiles of JMJD3 knocked down GM-BMDMs (55), IL6 was identified as a top upstream regulator and its downregulated expression level could contribute to the changes of its downstream gene expression (**Figure 6I**). Besides various immune related pathways, cell survival related pathways were also downregulated in M-BMDMs transfected with si-JMJD3 comparing with their GM-BMDMs counterparts (**Figure 6J**). All these results indicated JMJD3 is the major contributor in the H3K27me3 mediated transcription repression of IL6.

## Discussion

The strategic role of macrophage in RA has been described and discussed in detail (56–58). Macrophages have been characterized to be the key producers of cytokines relevant to the physiopathology of RA, such as IL6. These cytokines in turn promote local inflammation and induce osteoclast differentiation and further destruct the cartilage and bone (56). As the major macrophage growth and differentiation factor, the roles of GM-CSF have long been studied in the regulation of polarization and gene expression in macrophages, as well as in the pathogenesis of RA (59, 60). During the process of RA, GM-CSF is produced in large quantity and concentrated at the site of the lesion, as a result, macrophages are more inclined to differentiate into GM-BMDM phenotype. GM-CSF deficiency has been shown to protect mice from CIA (61). Moreover, GM-CSF and its induced macrophage polarization have been suggested to be the potential therapeutic targets in RA, psoriatic arthritis and all types of undifferentiated arthritis in patients (62). Indeed, targeting GM-CSF or its receptor has been shown the sustained clinical responses in RA patients (63). In the current study, we found that GM-CSF derived macrophages has less H3K27me3 expression. Demethylase inhibitor GSK-J4 could enhance the level of H3K27me3, and decrease the levels of proinflammatory cytokines, not only in the cultured cell system, but also in the CIA mice model. Both local and systemic inflammation are reduced upon GSK-J4 treatment, which establishing a link between the H3K27me3 demethylases’ activity with GM-CSF as well as the collagen induced arthritis, suggested a potential application of targeting epigenetic modifiers in RA patients.

Recent years, monoclonal antibody drugs against IL6 or its receptors have been developed as new methods for RA treatment in clinic, such as Sarilumab and Olokizumab (64–67). In the CIA model mice, we found that IL6 production was mainly from macrophage populations. Therefore, we proposed that reversing the H3K27me3-IL6 axis activity in macrophages may play a significant role in the process of RA. Furthermore, plenty of GM-CSF in RA lesion tends to divide macrophage into proinflammatory phenotype, and that matches with higher IL6 expression in our *in vitro* studies. Besides IL6, multiple proinflammatory cytokines, such as TNFα, IL1β and GM-CSF itself were identified to be regulated by H3K27me3. Enhancing levels of H3K27me3 by GSK-J4 treatment could inhibit the expression of these crucial cytokines, which further contribute to the relief of RA symptoms. In addition, Cribbs observed GSK-J4 induced suppression of IL17 expression and reduced Th17 cell proliferation (68). IL17 was suggested to increase IL6 transcription in turn (69), which might be another feedback mechanism that GSK-J4 regulates IL6 transcription. Finally, the EMSA and binding activity assay suggested that H3K27me3 inhibited NFκB binding to its consensus sequences in macrophages, and GSK-J4 also inhibited the activities of p52 and p50 subunits besides canonical p65, indicating that noncanonical pathway may also involve in the process of H3K27me3 dependent IL6 transcriptional inhibition.

H3K27me3 silences gene transcription through fasten nucleosome to chromosome so that transcription regulates sequence elements, such as promoter, enhancer, response element, etc., become hard to perform their functions. Laurens Kruidenier preliminary clarified that GSK-J4 could control TNFα transcription in 2012 (29). As the main demethylases of H3K27me3, most researchers consider them playing redundant roles in erasing H3K27me3 modification (70, 71). Takashi Satoh et al. showed that JMJD3 mediated H3K27 demethylation was crucial for regulating M-BMDMs development process, but dispensable for GM-BMDMs (72). Cao’s research provided the evidence that UTX epigenetically promotes IL6 and IFNβ production in mice peritoneal macrophages (73). However, JMJD3, but not UTX, was shown to play a critical role in axial pattern formation in mice (74). In the current study, we observed that the expression of both UTX and JMJD3 was higher in M-BMDMs. EZH2 and UTX/JMJD3 modified H3K27 methylation and demethylation become more active in M-BMDMs comparing with GM-BMDMs. Both of UTX and JMJD3 contribute to IL6 transcriptional regulation, whereas JMJD3 but not UTX plays dominant roles in cell survival. JMJD3 was associated with apoptosis in osteoblast differentiation (75, 76) or stroke (57), however no obviously different pre-caspase3 and Bcl2 expression in GSK-J4 treated M-BMDMs and GM-BMDMs. Takashi Satoh suggested that JMJD3 was required for the cell-cycle progression of M-BMDMs (72), which may explain the phenomenon we observed, however more detailed work is needed for fully elucidating the specific roles of UTX and JMJD3 in the settings of H3K27me3 regulation, as well as in cell proliferation and survival.

## Data Availability Statement

The datasets presented in this study can be found in online repositories. The names of the repository/repositories and accession number(s) can be found below: https://www.ncbi.nlm.nih.gov/geo/, GSE188615.

## Ethics Statement

The animal study was reviewed and approved by Committee of Experimental Animals of the Ocean University of China.

## Author Contributions

ZZ, CZ, and JY designed research. ZZ, YZZ, and DG performed research. ZZ, WH, and QS contributed reagents/analytic tools. ZZ and XX analyzed data. ZZ, CZ, and JY wrote the paper. All authors contributed to the article and approved the submitted version.

## Funding

This work was supported by Key R&D Program of Shandong Province (2020CXGC010503) and Shandong Provincial Major Science and Technology Innovation Project (2018SDKJ0402).

## Conflict of Interest

The authors declare that the research was conducted in the absence of any commercial or financial relationships that could be construed as a potential conflict of interest.

## Publisher’s Note

All claims expressed in this article are solely those of the authors and do not necessarily represent those of their affiliated organizations, or those of the publisher, the editors and the reviewers. Any product that may be evaluated in this article, or claim that may be made by its manufacturer, is not guaranteed or endorsed by the publisher.

## References

[1] Smolen JS Aletaha D Barton A Burmester GR Emery P Firestein GS. Rheumatoid Arthritis. Nat Rev Dis Primers (2018) 4:18001. doi: 10.1038/nrdp.2018.1 29417936

[2] Boutet MA Courties G Nerviani A Le Goff B Apparailly F Pitzalis C. Novel Insights Into Macrophage Diversity in Rheumatoid Arthritis Synovium. Autoimmun Rev (2021) 20(3):102758. doi: 10.1016/j.autrev.2021.102758 33476818

[3] Yang X Li S Zhao Y Li S Zhao T Tai Y. GRK2 Mediated Abnormal Transduction of PGE2-EP4-cAMP-CREB Signaling Induces the Imbalance of Macrophages Polarization in Collagen-Induced Arthritis Mice. Cells (2019) 8(12):1–18. doi: 10.3390/cells8121596 PMC6953022 31818003

[4] Lacey DC Achuthan A Fleetwood AJ Dinh H Roiniotis J Scholz GM. Defining GM-CSF- and Macrophage-CSF-Dependent Macrophage Responses by *In Vitro* Models. J Immunol (2012) 188(11):5752–65. doi: 10.4049/jimmunol.1103426 22547697

[5] Jaguin M Houlbert N Fardel O Lecureur V. Polarization Profiles of Human M-CSF-Generated Macrophages and Comparison of M1-Markers in Classically Activated Macrophages From GM-CSF and M-CSF Origin. Cell Immunol (2013) 281(1):51–61. doi: 10.1016/j.cellimm.2013.01.010 23454681

[6] Hirota K Hashimoto M Ito Y Matsuura M Ito H Tanaka M. Autoimmune Th17 Cells Induced Synovial Stromal and Innate Lymphoid Cell Secretion of the Cytokine GM-CSF to Initiate and Augment Autoimmune Arthritis. Immunity (2018) 48(6):1220–32.e5. doi: 10.1016/j.immuni.2018.04.009 29802020 PMC6024031

[7] Lewis MJ Barnes MR Blighe K Goldmann K Rana S Hackney JA. Molecular Portraits of Early Rheumatoid Arthritis Identify Clinical and Treatment Response Phenotypes. Cell Rep (2019) 28(9):2455–70.e5. doi: 10.1016/j.celrep.2019.07.091 31461658 PMC6718830

[8] Li WC Bai L Xu Y Chen H Ma R Hou WB. Identification of Differentially Expressed Genes in Synovial Tissue of Rheumatoid Arthritis and Osteoarthritis in Patients. J Cell Biochem (2019) 120(3):4533–44. doi: 10.1002/jcb.27741 30260019

[9] Kuznetsova T Prange KHM Glass CK de Winther MPJ. Transcriptional and Epigenetic Regulation of Macrophages in Atherosclerosis. Nat Rev Cardiol (2020) 17(4):216–28. doi: 10.1038/s41569-019-0265-3 PMC7770754 31578516

[10] Kouzarides T. Chromatin Modifications and Their Function. Cell (2007) 128(4):693–705. doi: 10.1016/j.cell.2007.02.005 17320507

[11] Allis CD Jenuwein T. The Molecular Hallmarks of Epigenetic Control. Nat Rev Genet (2016) 17(8):487–500. doi: 10.1038/nrg.2016.59 27346641

[12] Saeed S Quintin J Kerstens HHD Rao NA Aghajanirefah A Matarese F. Epigenetic Programming of Monocyte-to-Macrophage Differentiation and Trained Innate Immunity. Science (2014) 345(6204):1251086–1–11. doi: 10.1126/science.1251086 PMC4242194 25258085

[13] De Santa F Narang V Yap ZH Tusi BK Burgold T Austenaa L. Jmjd3 Contributes to the Control of Gene Expression in LPS-Activated Macrophages. EMBO J (2009) 28(21):3341–52. doi: 10.1038/emboj.2009.271 PMC2752025 19779457

[14] Barski A Cuddapah S Cui K Roh TY Schones DE Wang Z. High-Resolution Profiling of Histone Methylations in the Human Genome. Cell (2007) 129(4):823–37. doi: 10.1016/j.cell.2007.05.009 17512414

[15] Li X Zhang Q Shi Q Liu Y Zhao K Shen Q. Demethylase Kdm6a Epigenetically Promotes IL-6 and IFN-Beta Production in Macrophages. J Autoimmun (2017) 80:85–94. doi: 10.1016/j.jaut.2017.02.007 28284523

[16] Parmar N Chandrakar P Kar S. Leishmania Donovani Subverts Host Immune Response by Epigenetic Reprogramming of Macrophage M(Lipopolysaccharides + IFN-Gamma)/M(IL-10) Polarization. J Immunol (2020) 204(10):2762–78. doi: 10.4049/jimmunol.1900251 32277055

[17] Zhang X Wang Y Yuan J Li N Pei S Xu J. Macrophage/microglial Ezh2 Facilitates Autoimmune Inflammation Through Inhibition of Socs3. J Exp Med (2018) 215(5):1365–82. doi: 10.1084/jem.20171417 PMC5940261 29626115

[18] Hong S Cho YW Yu LR Yu H Veenstra TD Ge K. Identification of JmjC Domain-Containing UTX and JMJD3 as Histone H3 Lysine 27 Demethylases. Proc Natl Acad Sci U S A (2007) 104(47):18439–44. doi: 10.1073/pnas.0707292104 PMC2141795 18003914

[19] Fan H Lu J Guo Y Li D Zhang ZM Tsai YH. BAHCC1 Binds H3K27me3 *via* a Conserved BAH Module to Mediate Gene Silencing and Oncogenesis. Nat Genet (2020) 52(12):1384–96. doi: 10.1038/s41588-020-00729-3 PMC8330957 33139953

[20] Cao Z Shi X Tian F Fang Y Wu JB Mrdenovic S. KDM6B is an Androgen Regulated Gene and Plays Oncogenic Roles by Demethylating H3K27me3 at Cyclin D1 Promoter in Prostate Cancer. Cell Death Dis (2021) 12(1):2. doi: 10.1038/s41419-020-03354-4 33414463 PMC7791132

[21] Morozov VM Li Y Clowers MM Ishov AM. Inhibitor of H3K27 Demethylase JMJD3/UTX GSK-J4 is a Potential Therapeutic Option for Castration Resistant Prostate Cancer. Oncotarget (2017) 8:62131–42. doi: 10.18632/oncotarget.19100 PMC5617492 28977932

[22] Yapp C Carr AJ Price A Oppermann U Snelling SJ. H3K27me3 Demethylases Regulate *In Vitro* Chondrogenesis and Chondrocyte Activity in Osteoarthritis. Arthritis Res Ther (2016) 18(1):158. doi: 10.1186/s13075-016-1053-7 27388528 PMC4936015

[23] Jiang Y Xiang C Zhong F Zhang Y Wang L Zhao Y. Histone H3K27 Methyltransferase EZH2 and Demethylase JMJD3 Regulate Hepatic Stellate Cells Activation and Liver Fibrosis. Theranostics (2021) 11(1):361–78. doi: 10.7150/thno.46360 PMC7681085 33391480

[24] Malinczak CA Rasky AJ Fonseca W Schaller MA Allen RM Ptaschinski C. Upregulation of H3K27 Demethylase KDM6 During Respiratory Syncytial Virus Infection Enhances Proinflammatory Responses and Immunopathology. J Immunol (2020) 204(1):159–68. doi: 10.4049/jimmunol.1900741 PMC6920528 31748348

[25] Connor MG Camarasa TMN Patey E Rasid O Barrio L Weight CM. The Histone Demethylase KDM6B Fine-Tunes the Host Response to Streptococcus Pneumoniae. Nat Microbiol (2021) 6(2):257–69. doi: 10.1038/s41564-020-00805-8 33349663

[26] Wu W Qin M Jia W Huang Z Li Z Yang D. Cystathionine-Gamma-Lyase Ameliorates the Histone Demethylase JMJD3-Mediated Autoimmune Response in Rheumatoid Arthritis. Cell Mol Immunol (2019) 16(8):694–705. doi: 10.1038/s41423-018-0037-8 29844591 PMC6804949

[27] Neele AE Gijbels MJJ van der Velden S Hoeksema MA Boshuizen MCS Prange KHM. Myeloid Kdm6b Deficiency Results in Advanced Atherosclerosis. Atherosclerosis (2018) 275:156–65. doi: 10.1016/j.atherosclerosis.2018.05.052 29908485

[28] Neele AE Prange KH Hoeksema MA v. d. Velden S Lucas T Dimmeler S. Macrophage Kdm6b Controls the Profibrotic Transcriptome Signature of Foam Cells. Epigenomics (2017) 4:383–91. doi: 10.2217/epi-2016-0152 28322580

[29] Kruidenier L Chung CW Cheng Z Liddle J Che K Joberty G. A Selective Jumonji H3K27 Demethylase Inhibitor Modulates the Proinflammatory Macrophage Response. Nature (2012) 488(7411):404–8. doi: 10.1038/nature11262 PMC4691848 22842901

[30] Li Y Zhang M Sheng M Zhang P Chen Z Xing W. Therapeutic Potential of GSK-J4, a Histone Demethylase KDM6B/JMJD3 Inhibitor, for Acute Myeloid Leukemia. J Cancer Res Clin Oncol (2018) 144(6):1065–77. doi: 10.1007/s00432-018-2631-7 PMC5948279 29594337

[31] Chu X Zhong L Yu L Xiong L Li J Dan W. GSK-J4 Induces Cell Cycle Arrest and Apoptosis *via* ER Stress and the Synergism Between GSK-J4 and Decitabine in Acute Myeloid Leukemia KG-1a Cells. Cancer Cell Int (2020) 20:209. doi: 10.1186/s12935-020-01297-6 32514253 PMC7268296

[32] Lobo-Silva J Cabral FJ Amaral MS Miyasato PA de Freitas RP Pereira ASA. The Antischistosomal Potential of GSK-J4, an H3K27 Demethylase Inhibitor: Insights From Molecular Modeling, Transcriptomics and *In Vitro* Assays. Parasit Vectors (2020) 13(1):140. doi: 10.1186/s13071-020-4000-z 32178714 PMC7077139

[33] Donas C Neira J Osorio-Barrios F Carrasco M Fernandez D Prado C. The Demethylase Inhibitor GSK-J4 Limits Inflammatory Colitis by Promoting *De Novo* Synthesis of Retinoic Acid in Dendritic Cells. Sci Rep (2021) 11(1):1342. doi: 10.1038/s41598-020-79122-3 33446666 PMC7809056

[34] Nakano K Whitaker JW Boyle DL Wang W Firestein GS. DNA Methylome Signature in Rheumatoid Arthritis. Ann Rheum Dis (2013) 72(1):110–7. doi: 10.1136/annrheumdis-2012-201526 PMC3549371 22736089

[35] Zhang L Pavicic PG Datta S Song Q Xu X Wei W. Unfolded Protein Response Differentially Regulates TLR4-Induced Cytokine Expression in Distinct Macrophage Populations. Front Immunol (2019) 10:1390. doi: 10.3389/fimmu.2019.01390 31293572 PMC6598306

[36] Zhao Z Zhang L Guo XD Cao LL Xue TF Zhao XJ. Rosiglitazone Exerts an Anti-Depressive Effect in Unpredictable Chronic Mild-Stress-Induced Depressive Mice by Maintaining Essential Neuron Autophagy and Inhibiting Excessive Astrocytic Apoptosis. Front Mol Neurosci (2017) 10:293. doi: 10.3389/fnmol.2017.00293 28959186 PMC5603714

[37] Nan J Wang Y Yang J Stark GR. IRF9 and Unphosphorylated STAT2 Cooperate With NF-κb to Drive IL6 Expression. Proc Natl Acad Sci U S A (2018) 115(15):3906–11. doi: 10.1073/pnas.1714102115 PMC5899435 29581268

[38] Wang Y Song Q Huang W Lin Y Wang X Wang C. A Virus-Induced Conformational Switch of STAT1-STAT2 Dimers Boosts Antiviral Defenses. Cell Res (2020) 31:206–18. doi: 10.1038/s41422-020-0386-6 PMC7405385 32759968

[39] Aletaha D Smolen JS. Diagnosis and Management of Rheumatoid Arthritis: A Review. JAMA (2018) 320(13):1360–72. doi: 10.1001/jama.2018.13103 30285183

[40] Pandolfi F Franza L Carusi V Altamura S Andriollo G Nucera E. Interleukin-6 in Rheumatoid Arthritis. Int J Mol Sci (2020) 21(15):5238. doi: 10.3390/ijms21155238 PMC7432115 32718086

[41] Glasson SS Chambers MG Van Den Berg WB Little CB. The OARSI Histopathology Initiative - Recommendations for Histological Assessments of Osteoarthritis in the Mouse. Osteoarthritis Cartilage (2010) 18 Suppl:3, S17–23. doi: 10.1016/j.joca.2010.05.025 20864019

[42] Yang P Qian FY Zhang MF Xu AL Wang X Jiang BP. Th17 Cell Pathogenicity and Plasticity in Rheumatoid Arthritis. J Leukocyte Biol (2019) 106(6):1233–40. doi: 10.1002/jlb.4ru0619-197r 31497905

[43] Yang J Sundrud MS Skepner J Yamagata T. Targeting Th17 Cells in Autoimmune Diseases. Trends Pharmacol Sci (2014) 35(10):493–500. doi: 10.1016/j.tips.2014.07.006 25131183

[44] Yan L Liang M Yang T Ji J Jose Kumar GS Sreena Hou X. The Immunoregulatory Role of Myeloid-Derived Suppressor Cells in the Pathogenesis of Rheumatoid Arthritis. Front Immunol (2020) 11:568362:568362. doi: 10.3389/fimmu.2020.568362 33042149 PMC7522347

[45] Boissier MC Assier E Biton J Denys A Falgarone G Bessis N. Regulatory T Cells (Treg) in Rheumatoid Arthritis. Joint Bone Spine (2009) 76(1):10–4. doi: 10.1016/j.jbspin.2008.08.002 19028128

[46] Komatsu N Okamoto K Sawa S Nakashima T Oh-hora M Kodama T. Pathogenic Conversion of Foxp3+ T Cells Into TH17 Cells in Autoimmune Arthritis. Nat Med (2014) 20(1):62–8. doi: 10.1038/nm.3432 24362934

[47] Shapouri-Moghaddam A Mohammadian S Vazini H Taghadosi M Esmaeili S-A Mardani F. Macrophage Plasticity, Polarization, and Function in Health and Disease. J Cell Physiol (2018) 233(9):6425–40. doi: 10.1002/jcp.26429 PMC13482560 29319160

[48] Fleetwood AJ Dinh H Cook AD Hertzog PJ Hamilton JA. GM-CSF- and M-CSF-Dependent Macrophage Phenotypes Display Differential Dependence on Type I Interferon Signaling. J Leukoc Biol (2009) 86(2):411–21. doi: 10.1189/jlb.1108702 19406830

[49] Martinez FO Gordon S. The M1 and M2 Paradigm of Macrophage Activation: Time for Reassessment. F1000Prime Rep (2014) 6:13. doi: 10.12703/P6-13 24669294 PMC3944738

[50] Murray PJ Allen JE Biswas SK Fisher EA Gilroy DW Goerdt S. Macrophage Activation and Polarization: Nomenclature and Experimental Guidelines. Immunity (2014) 41(1):14–20. doi: 10.1016/j.immuni.2014.06.008 25035950 PMC4123412

[51] Lukic A Larssen P Fauland A Samuelsson B Wheelock CE Gabrielsson S. GM-CSF- and M-CSF-Primed Macrophages Present Similar Resolving But Distinct Inflammatory Lipid Mediator Signatures. FASEB J (2017) 31(10):4370–81. doi: 10.1096/fj.201700319R 28637652

[52] Patel U Rajasingh S Samanta S Cao T Dawn B Rajasingh J. Macrophage Polarization in Response to Epigenetic Modifiers During Infection and Inflammation. Drug Discovery Today (2017) 22(1):186–93. doi: 10.1016/j.drudis.2016.08.006 PMC5226865 27554801

[53] Yin X Yang S Zhang M Yue Y. The Role and Prospect of JMJD3 in Stem Cells and Cancer. BioMed Pharmacother (2019) 118:109384. doi: 10.1016/j.biopha.2019.109384 31545292

[54] Samanta S Zhou Z Rajasingh S Panda A Sampath V Rajasingh J. DNMT and HDAC Inhibitors Together Abrogate Endotoxemia Mediated Macrophage Death by STAT3-JMJD3 Signaling. Int J Biochem Cell Biol (2018) 102:117–27. doi: 10.1016/j.biocel.2018.07.002 PMC6309960 30010012

[55] Kramer A Green J Pollard J Jr. Tugendreich S. Causal Analysis Approaches in Ingenuity Pathway Analysis. Bioinformatics (2014) 30(4):523–30. doi: 10.1093/bioinformatics/btt703 PMC3928520 24336805

[56] Udalova IA Mantovani A Feldmann M. Macrophage Heterogeneity in the Context of Rheumatoid Arthritis. Nat Rev Rheumatol (2016) 12(8):472–85. doi: 10.1038/nrrheum.2016.91 27383913

[57] Zhang H Wang J Huang J Shi T Ma X Luo X. Inhibiting Jumoji Domain Containing Protein 3 (JMJD3) Prevent Neuronal Apoptosis From Stroke. Exp Neurol (2018) 308:132–42. doi: 10.1016/j.expneurol.2018.07.007 30028997

[58] Yang Y Guo L Wang Z Liu P Liu X Ding J. Targeted Silver Nanoparticles for Rheumatoid Arthritis Therapy *via* Macrophage Apoptosis and Re-Polarization. Biomaterials (2021) 264:120390. doi: 10.1016/j.biomaterials.2020.120390 32980634

[59] Lotfi N Thome R Rezaei N Zhang GX Rezaei A Rostami A. Roles of GM-CSF in the Pathogenesis of Autoimmune Diseases: An Update. Front Immunol (2019) 10:1265. doi: 10.3389/fimmu.2019.01265 31275302 PMC6593264

[60] Liu W Zhang Y Zhu W Ma C Ruan J Long H. Sinomenine Inhibits the Progression of Rheumatoid Arthritis by Regulating the Secretion of Inflammatory Cytokines and Monocyte/Macrophage Subsets. Front Immunol (2018) 9:2228:2228. doi: 10.3389/fimmu.2018.02228 30319663 PMC6168735

[61] Campbell IK Rich MJ Bischof RJ Dunn AR Grail D Hamilton JA. Protection From Collagen-Induced Arthritis in Granulocyte-Macrophage Colony-Stimulating Factor-Deficient Mice. J Immunol (1998) 161(17):3639–44. 9759887

[62] Fuentelsaz-Romero S Cuervo A Estrada-Capetillo L Celis R Garcia-Campos R Ramirez J. GM-CSF Expression and Macrophage Polarization in Joints of Undifferentiated Arthritis Patients Evolving to Rheumatoid Arthritis or Psoriatic Arthritis. Front Immunol (2020) 11:613975:613975. doi: 10.3389/fimmu.2020.613975 33679701 PMC7925849

[63] Wu X. Innate Lymphocytes in Inflammatory Arthritis. Front Immunol (2020) 11:565275:565275. doi: 10.3389/fimmu.2020.565275 33072104 PMC7544949

[64] Kerschbaumer A Sepriano A Smolen JS v. d. Heijde D v. V. Maxime Dougados R McInnes IB. Efficacy of Pharmacological Treatment in Rheumatoid Arthritis: A Systematic Literature Research Informing the 2019 Update of the EULAR Recommendations for Management of Rheumatoid Arthritis. Ann Rheum Dis (2020) 79:744–59. doi: 10.1136/annrheumdis-2019-216656 PMC7286044 32033937

[65] Burmeste GR Lin Y Patel R v. Adelsberg J Mangan EK Graham NMH. Efficacy and Safety of Sarilumab Monotherapy Versus Adalimumab Monotherapy for the Treatment of Patients With Active Rheumatoid Arthritis (MONARCH): A Randomised, Double-Blind, Parallel-Group Phase III Trial. Ann Rheum Dis (2017) 76:840–7. doi: 10.1136/annrheumdis-2016-210310 PMC5530335 27856432

[66] Lamb YN Deeks ED. Sarilumab: A Review in Moderate to Severe Rheumatoid Arthritis. Drugs (2018) 78(9):929–40. doi: 10.1007/s40265-018-0929-z 29931592

[67] Behrens F Tak PP Ostergaard M Stoilov R Wiland P Huizinga TW. MOR103, a Human Monoclonal Antibody to Granulocyte-Macrophage Colony-Stimulating Factor, in the Treatment of Patients With Moderate Rheumatoid Arthritis: Results of a Phase Ib/IIa Randomised, Double-Blind, Placebo-Controlled, Dose-Escalation Trial. Ann Rheum Dis (2015) 74(6):1058–64. doi: 10.1136/annrheumdis-2013-204816 PMC4431325 24534756

[68] Cribbs AP Terlecki-Zaniewicz S Philpott M Baardman J Ahern D Lindow M. Histone H3K27me3 Demethylases Regulate Human Th17 Cell Development and Effector Functions by Impacting on Metabolism. Proc Natl Acad Sci U S A (2019) 117:6056–66. doi: 10.1073/pnas.1919893117 PMC7084125 32123118

[69] Miossec P Kolls JK. Targeting IL-17 and TH17 Cells in Chronic Inflammation. Nat Rev Drug Discov (2012) 11(10):763–76. doi: 10.1038/nrd3794 23023676

[70] Berger SL. The Complex Language of Chromatin Regulation During Transcription. Nature (2007) 447(7143):407–12. doi: 10.1038/nature05915 17522673

[71] Ferrari KJ Scelfo A Jammula S Cuomo A Barozzi I Stutzer A. Polycomb-Dependent H3K27me1 and H3K27me2 Regulate Active Transcription and Enhancer Fidelity. Mol Cell (2014) 53(1):49–62. doi: 10.1016/j.molcel.2013.10.030 24289921

[72] Satoh T Takeuchi O Vandenbon A Yasuda K Tanaka Y Kumagai Y. The Jmjd3-Irf4 Axis Regulates M2 Macrophage Polarization and Host Responses Against Helminth Infection. Nat Immunol (2010) 11(10):936–44. doi: 10.1038/ni.1920 20729857

[73] Donas C Carrasco M Fritz M Prado C Tejon G Osorio-Barrios F. The Histone Demethylase Inhibitor GSK-J4 Limits Inflammation Through the Induction of a Tolerogenic Phenotype on DCs. J Autoimmun (2016) 75:105–17. doi: 10.1016/j.jaut.2016.07.011 27528513

[74] Naruse C Shibata S Tamura M Kawaguchi T Abe K Sugihara K. New Insights Into the Role of Jmjd3 and Utx in Axial Skeletal Formation in Mice. FASEB J (2017) 31(6):2252–66. doi: 10.1096/fj.201600642R 28188179

[75] Yang D Yu B Sun H Qiu L. The Roles of Histone Demethylase Jmjd3 in Osteoblast Differentiation and Apoptosis. J Clin Med (2017) 6(3):1–5. doi: 10.3390/jcm6030024 PMC5372993 28241471

[76] Sun HY Yang D Mi J Yu YQ Qiu LH. Histone Demethylase Jmjd3 Modulates Osteoblast Apoptosis Induced by Tumor Necrosis Factor-Alpha Through Directly Targeting RASSF5. Connect Tissue Res (2020) 61(6):517–25. doi: 10.1080/03008207.2019.1620225 31092054

