# Supplementary material for: Inhibition of Histone H3 Lysine-27 Demethylase Activity Relieves Rheumatoid Arthritis Symptoms via Repression of IL6 Transcription in Macrophages
Source: Front Immunol. 2022 Mar 15;13:818070. doi: 10.3389/fimmu.2022.818070 (PMC8965057; doi:10.3389/fimmu.2022.818070)
Supplement: Supplementary file 1 [file DataSheet_1.doc]

**Supplemental Figures**


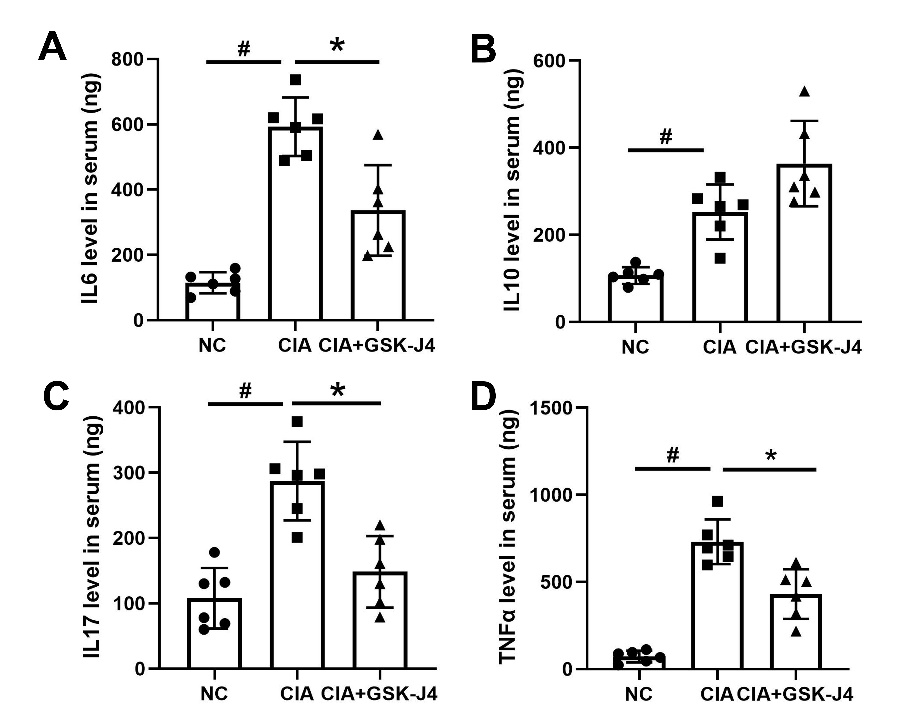


**Figure S1**. IL6, IL10, IL17 and TNFα concentration in peripheral blood of CIA and GSK-J4 treated mice. Error bars indicate SD, p < 0.05 (two-tailed Student's T-test) is indicated by * for comparison with CIA vs CIA+GSK-J4, # for comparison with NC vs CIA.


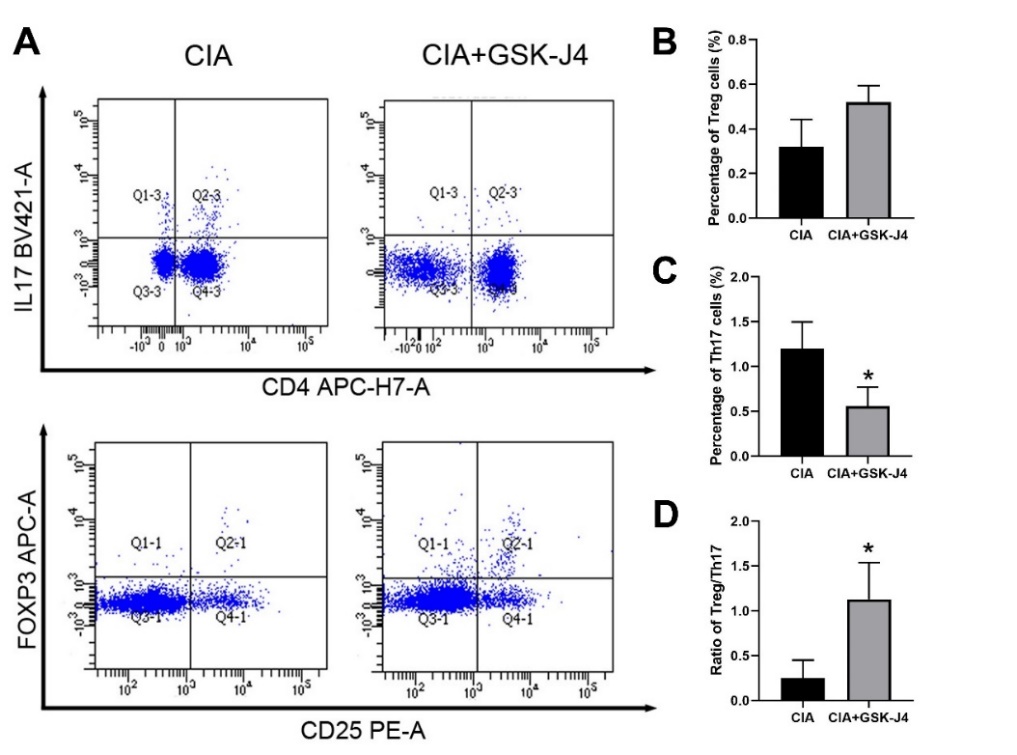


**Figure S2**. (A) Flow cytometry detected CD4^+^IL17^+^ (Th17 cells) and CD25^+^FOXP3^+^ (Treg cells) cell abundances in CIA and GSK-J4 treatment mice [inguinal](javascript:;) lymph nodes. (B to D) The percentage of Treg and Th17 cells and the ratio of Treg/Th17 were calculated. * p < 0.05 (two-tailed Student's T-test), error bars indicate SD. * for comparison with CIA vs CIA+GSK-J4.


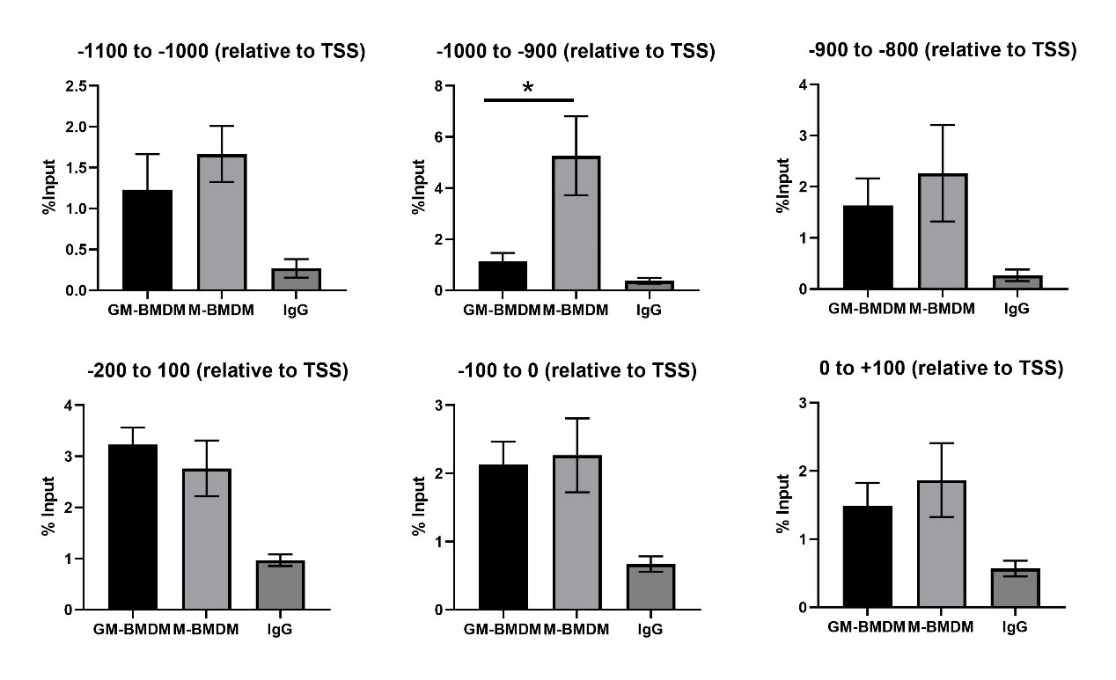


**Figure S3.** ChIP analysis of H3K27me3 binding in different regions upstream of the IL6 transcription start site. There is a significant DNA enrichment between the -1000 to -900 bps region. Error bars indicate SD, p < 0.05 (two-tailed Student's T-test) is indicated by * for comparison with GM-BMDM vs M-BMDM.


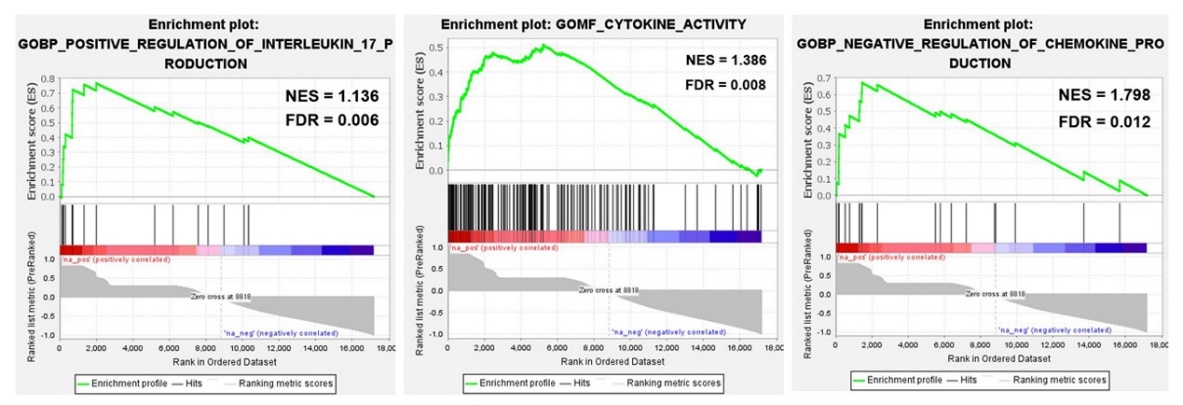


**Figure S4**. Enrichment plot ranked the top2 to 4 were shown by GSEA in the gene matrix downloaded from [*ftp.broadinstitute.org://pub/gsea/gene_sets/c5.bp.v6.0.symbols.gmt*](ftp://ftp.broadinstitute.org://pub/gsea/gene_sets/c5.bp.v6.0.symbols.gmt).


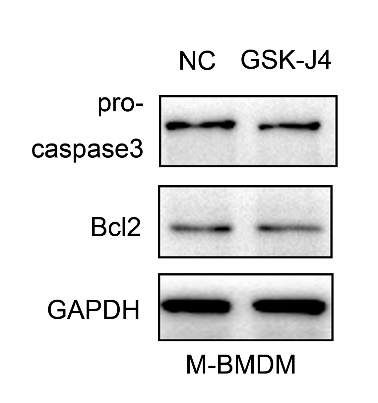


**Figure S5**. GSK-J4 administration couldn’t induce apoptosis related protein change. Western blot analyzed pro-caspase3 and Bcl2 expression changes of GSK-J4 treated M-BMDMs.
